# Howe Duality and Dynamical Weyl Group

**DOI:** 10.1007/s00031-024-09888-7

**Published:** 2024-12-06

**Authors:** Rea Dalipi, Giovanni Felder, Anfisa Gurenkova

**Affiliations:** 1 Department of Mathematics, University of Geneva, Rue du Conseil-Général 7-9, 1205 Geneva, Switzerland; 2 Department of Mathematics, ETH Zurich, 8092 Zurich, Switzerland; 3 Krichever Center for Advanced Studies, Skolkovo Institute of Science and Technology, Moscow, Russia

**Keywords:** Quantum groups, Howe duality, Dynamical Weyl group, Primary 17B37, Secondary 16T25

## Abstract

We give a fermionic formula for *R*-matrices of exterior powers of the vector representations of $U_q(\widehat{ \mathfrak {gl}}_N)$ and relate it to the dynamical Weyl group of Tarasov–Varchenko and Etingof–Varchenko, via a Howe ($\mathfrak {gl}_N,\mathfrak {gl}_M)$-duality. In the limit $N\rightarrow \infty$ we obtain *R*-matrices for Fock spaces. As a consequence of our result we obtain a dynamical action of the Weyl group on integrable $U_q\mathfrak {gl}_M$-modules, extending the known action on zero weight spaces. In an Appendix by Anfisa Gurenkova it is shown that the latter property also holds if we replace $\mathfrak {gl}_M$ by a general symmetrizable Kac–Moody algebra.

## Introduction

The symmetric groups play two different roles in the representation theory of general linear groups. On one side, let us call it the Weyl side, the symmetric group $$S_N$$ of permutations of *N* letters is the subgroup of $$ GL _N$$ of permutation matrices and thus acts on any representation of $$ GL _N$$. On the other side, say the Schur side, $$S_M$$ is the group of permutation of factors of the *M*th tensor power $$U^{\otimes M}=U\otimes \cdots \otimes U$$ of a representation *U* of $$ GL _N$$ and the action of $$S_M$$ commutes with the natural action of $$ GL _N$$ on $$U^{\otimes M}$$. We will focus on the case where *U* is an exterior power of the vector representation, but let us be general for the time being.

Both roles admit a *q*-deformed version but the symmetric group needs to be replaced by the braid group. On the Weyl side one has an action of the braid group $B_N$ on finite dimensional representations of the quantum enveloping algebras $U_{q} \mathfrak {gl}_{N}$ deforming the above action of the symmetric group. This is a special case of the action on integrable representations of the Artin braid group corresponding to the Weyl group of any semisimple Lie algebra [15, 16]. On the Schur side, the universal *R*-matrix of $U_q\mathfrak gl_N$ defines a representation of the braid group $B_M$ on the tensor power $U^{\otimes M}$ of a finite dimensional representation *U* of $U_q{\mathfrak {gl}}_N$. The action of the generator $s_i$, braiding the *i*th with the $i+1$st strand is given by the action of the *R*-matrix on the *i*th and $i+1$st factor.

Both sides are degenerate cases of versions with parameters, called spectral (or equivariant) parameters on the Schur side and dynamical (or Kähler) parameters on the Weyl side. In terms of representation theory, this generalization amounts to replacing $U_q\mathfrak {gl}_N$ by its affine version $U_q\widehat{\mathfrak {gl}}_N$. A feature of the versions with parameters, whose clarification is a goal of this paper, is that the symmetric group plays the leading role, rather than the braid group that is relevant in the degenerate limit. The *R*-matrices of quantum groups first appeared in statistical mechanics and quantum field theory as solutions $R(z)\in {\text {End}}(U\otimes U)$ of the Yang–Baxter equation, depending meromorphically on a complex spectral parameter *z*. The Yang–Baxter equation is equivalent to the braiding relation (4) for the braiding matrix $\check{R}(z)=P\circ R(z)$ obtained by composing the *R*-matrix with the permutation $P(u\otimes v)=v\otimes u$ of factors. Moreover one has the inversion (or unitarity) relation $\check{R}(z^{-1})\check{R}(z)=\textrm{id}$. One obtains, on the Schur side, a representation of the symmetric group $S_M$ on $U^{\otimes M}$-valued meromorphic functions $f(z_1,\dots , z_M)$ of *M* variables, such that the generator $s_i=(i,i+1)$ is mapped to

$$ s_if(z_1,\dots ,z_N) = \text {id}^{\otimes (i-1)}\otimes \check{R}(z_i/z_{i+1})\otimes \text {id}^{\otimes (N-i-1)} f(z_1,\dots ,z_{i+1},z_i,\dots ,z_N). $$

Representations of the braid group on $U^{\otimes M}$ appear in the limits $z_i/z_{i+1}\rightarrow 0$, $i=1,\dots ,M-1$ or $\infty$. Indeed *R*(*z*) converges in the limit $z\rightarrow \infty$ to the solution of the Yang–Baxter equation without spectral parameter corresponding to $U_q{\mathfrak {gl}}_N$. The relation $s_i^2=1$ is lost in this limit and one is left with a representation of the braid group.

On the Weyl side, the discovery of a version of the quantum Weyl group action with parameters came later [7, 24] and is called dynamical Weyl group. More recently [20] a geometric realization of a dynamical Weyl group action on the equivariant *K*-theory of Nakajima varieties was found. Let $T\subset SL_M(\mathbb {C})$ be the Cartan torus of diagonal matrices, $P_M={\text {Hom}}(T,\mathbb {C}^\times )$ the weight lattice. Then finite dimensional representations *U* of $U_q\mathfrak {sl}_M$ (of type I with *q* not a root of unity) have a weight decomposition $U=\oplus _{\mu \in P_M}U[\mu ]$. The Weyl group $S_M$ acts on *T* and $P_M$, defining a action of the braid group via the canonical homomorphism $B_M\rightarrow S_M$. A dynamical action of the braid group $B_M$ on an $\mathfrak {sl}_M$-module *U* is a representation $\rho$ of the braid group on rational *U*-valued function on *T* of the form

$$ (\rho (g)f)(t)=A_{g}(g^{-1}t)f(g^{-1}t), $$

for some rational functions $t\mapsto A_g(t)\in {\text {End}}(U)$, compatible with the *T*-action on *U* in the sense that $A_g(t)(U[\mu ])\subset U[g\mu ]$. The theory of intertwining operators developed in [7, 24], which works for arbitrary simple Lie algebras, gives rise to a dynamical action of the braid group with many interesting properties, such as universal formulas for $A_g(t)|_{U[\mu ]}$ as the action of an element of $U_q\mathfrak {sl}_M$ independent of *U* and a compatibility with inclusions of Lie subalgebras, implying that $A_{s_i}(t)$ for generators $s_i$ is obtained as the image of the corresponding element of $U_q(\mathfrak {sl}_2)$ by the inclusion associated with the *i*th simple root.

In this paper we take $$U=\bigwedge V$$, the direct sum of exterior powers of the vector representation $$V=\mathbb {C}^N$$ of $$U_q{\mathfrak {gl}}_N$$, and relate the two actions of the symmetric group via quantum (skew) Howe duality. The classical Howe duality [10] is based on the observation that on $$\bigwedge (\mathbb {C}^N\otimes \mathbb {C}^M)$$ we have natural commuting actions of $$ GL _N$$ and $$ GL _M$$ whose images span each other’s commutant in the endomorphism ring. This implies that endomorphisms of $$(\bigwedge V)^{\otimes M}=\bigwedge V\otimes \cdots \otimes \bigwedge V$$ commuting with the $$ GL_N $$ action on $$V=\mathbb {C}^N$$ are spanned by a commuting action of $$ GL _M$$. There is a also a symmetric version of Howe duality, where exterior powers are replaced by symmetric powers. Quantum version of Howe dualities are known. The case of symmetric powers was first observed and proved by Toledano Laredo in [27]. The skew-symmetric case was then considered in [3] and [14]. See [22] for recent developments of this story. In Section 3 we present an approach based on the action of the Clifford algebra which is more elementary than the categorification approach of [4], see Proposition 3.1.

In particular one obtains a $U_q{\mathfrak {gl}}_M$-action on $(\bigwedge V)^{\otimes M}$ commuting with the diagonal $U_q{\mathfrak {gl}}_N$-action. The upshot is that the action of the symmetric group on $(\bigwedge V)^{\otimes M}$-valued functions by braiding matrices with spectral parameter is realized by a dynamical Weyl group action for the $U_q{\mathfrak {gl}}_M$-action, after a suitable identification of spectral with dynamical parameters. Thus we connect the Schur with the Weyl side. In the limit of infinite spectral/dynamical parameter we recover the result of [3] relating braid group action with quantum Weyl group action.

For $M=2$ we obtain an explicit formula for the *R*-matrix for pairs of exterior powers of the vector representation in terms of $U_q{\mathfrak {gl}}_2$, see Theorem 2.6. These *R*-matrices were calculated in [6] by the fusion procedure and are expressed in terms of projections onto irreducible subrepresentations of the tensor product. Our alternative formula does not rely on the decomposition into irreducible subrepresentations. It is a sum of terms, each with a simple pole in the spectral parameters, and gives an interpretation of the residues at the poles. Also, it is given by a formula without explicit dependence on *N*. In fact it has a well-defined limit as $N\rightarrow \infty$ resulting in solutions of the Yang–Baxter equations for fermionic Fock spaces, a.k.a. spaces of semi-infinite exterior powers, see Theorem 2.8.

When written in terms of Clifford algebra generators, our formula for the *R*-matrix of $\bigwedge ^kV\otimes \bigwedge ^kV$ converges in the limit $q\rightarrow 1,z\rightarrow 1$ to the formula proposed by Smirnov [21] in the case of the Yangian, see Theorem 4.6. Finding a conceptual proof of Smirnov’s formula was an initial motivation of this work. Note also that similar constructions in this limit are known in the context of Knizhnik–Zamolodchikov and dynamical equations [17–19, 23, 25, 27, 28].

As an application of our result, we obtain in Theorem 4.1 and Corollary 4.2 a dynamical action of the *symmetric group* on integrable representations of $U_q{\mathfrak {gl}}_M$, rather than of the braid group as in [7]. In fact our dynamical action on functions with values in a representation *U* differs from the dynamical action of the braid group defined in [7] by a transformation $A_{g}(t)|_{U[\mu ]}\mapsto A_{g}(t)|_{U[\mu ]}f_{g,\mu }(t)$ for some scalar meromorphic functions $f_{g,\mu }(t)$ obeying the cocycle condition $f_{gh,\mu }(t)=f_{g,h\mu }(ht)f_{h,\mu }(t)$ for any braids *g*, *h*, see Proposition 4.3. While in [7] the dynamical action of the braid group factors through the symmetric group only when restricted to the zero weight space *U*[0], our modified action defines a representation of the symmetric group on the whole space of *U*-valued functions. In fact the same is true for general Kac–Moody algebras: our modified action defines an action of the Weyl group on functions with values in integrable representations, see Theorem 4.4. A proof is provided by Anfisa Gurenkova in Appendix B.

Part of the results of this paper were announced in the Oberwolfach report [8].

## Braiding Matrices for Exterior Powers of the Vector Representation

### *q*-number Notation

We adopt the standard *q*-number notation: for $n\in \mathbb {Z}_{\ge 0}$, $[n]_q=\frac{q^n-q^{-n}}{q-q^{-1}}$. The *q*-factorial is defined by $[0]_q!=1$, $[n]_q!=\prod _{j=1}^n[j]_q$, $n\ge 1$ and the *q*-binomial coefficient (or Gauss polynomial) is

$$ \bigg [\!\begin{array}{c} {n}\\ {j} \end{array}\!\bigg ]_{q}=\frac{[n]_q!}{[j]_q![n-j]_q!}. $$

All these objects belong to $\mathbb {Z}[q,q^{-1}]$.

### Quantum Enveloping Algebras of Type $A_{N-1}$ and $A_{N-1}^{(1)}$

Drinfeld–Jimbo quantum enveloping algebras are the subject of several textbooks, for example [5, 16]. Here we collect our conventions. Let *q* be a non-zero complex number which is not a root of unity. Let $(a_{ij})_{i,j=0}^{N-1}$ be the Cartan matrix of $A_{N-1}^{(1)}$: $a_{ij}=2$ if $i=j$, $a_{ij}=-1$ if $j=i\pm 1\mod N$, and $a_{ij}=0$ otherwise. It is convenient to identify the index set $\{0,\dots ,N-1\}$ with $\mathbb {Z}/N\mathbb {Z}$ in view of the symmetry $a_{i+1,j+1}=a_{ij}$. The Hopf algebra $U'_q(\widehat{\mathfrak {sl}}_N)$ is the unital algebra over $\mathbb {C}$ generated by $e_i,f_i,k_i^{\pm 1}$, $(i=0,\dots , N-1)$ and relations

$$\begin{aligned} k_ik_j&=k_jk_i,\quad k_ik_i^{-1}=k_i^{-1}k_i=1 \\ k_ie_jk_i^{-1}&=q^{a_{ij}}e_j,\quad k_if_jk_i^{-1}=q^{-a_{ij}}f_j, \\ e_if_j-f_je_i&=\delta _{ij}\frac{k_i-k_i^{-1}}{q-q^{-1}} \\ \sum _{r=0}^{1-a_{ij}}&\bigg [\!\begin{array}{c} {1-a_{ij}}\\ {r} \end{array}\!\bigg ]_{q} e_i^re_je_i^{1-a_{ij}-r} =0,\quad \text {if } i\ne j, \\ \sum _{r=0}^{1-a_{ij}}&\bigg [\!\begin{array}{c} {1-a_{ij}}\\ {r} \end{array}\!\bigg ]_{q} f_i^rf_jf_i^{1-a_{ij}-r} =0,\quad \text {if } i\ne j. \end{aligned}$$

The coproduct is defined on generators as $\Delta (e_i)=e_i\otimes 1+k_i\otimes e_i$, $\Delta (f_i)=f_i\otimes k_i^{-1}+ 1\otimes f_i$ and $\Delta (k^{\pm 1}_i)=k^{\pm 1}_i\otimes k^{\pm 1}_i$. The element $\prod _{i=0}^{N-1}k_i$ is central and group-like. For finite dimensional representations of $U_q'(\widehat{\mathfrak {sl}}_N)$ this central element acts by 1. The quantum loop algebra $U_qL{\mathfrak {sl}}_N$ is the quotient of $U'_q(\widehat{\mathfrak {sl}}_N)$ by the relation $\prod _{i=0}^{N-1}k_i=1$. The Hopf algebra structure descends to this quotient. It is a deformation of the universal enveloping algebra of the Lie algebra $L\mathfrak {sl}_N={\mathfrak {sl}}_N[t,t^{-1}]$ of loops in ${\mathfrak {sl}}_N$. The Hopf algebra $U_qL{\mathfrak {sl}}_N$ contains the Hopf subalgebra $U_q {\mathfrak {sl}}_N$ generated by $e_i,f_i,k_i$ with $1\le i\le N-1$.

For $k\in \mathbb {Z}_{\ge 0}$ the *k*th divided power of generators is defined as

$$ e_i^{(k)}=\frac{e_i^k}{[k]_q!},\quad f_i^{(k)}=\frac{f_i^k}{[k]_q!}, $$

with the convention that $e_i^{(0)}=f_i^{(0)}=1$.

Let $\mathbb {Z}^N=\oplus _{i\in \mathbb {Z}/N\mathbb {Z}}\mathbb {Z}\epsilon _i$ be the (co)weight lattice of ${\mathfrak {gl}}_N$ with standard inner product $(\ |\ )$, which identifies the Cartan subalgebra of diagonal matrix with its dual. The root lattice of vectors of zero coordinate sum is spanned by the simple roots $\alpha _i=\epsilon _i-\epsilon _{i+1}$, $(i=1,\dots , N-1)$. We also set $\alpha _0=\epsilon _N-\epsilon _1$, the highest root. The Hopf algebra $U_qL{\mathfrak {gl}}_N$ is obtained by adjoining generators $t_\lambda$ for $\lambda \in \mathbb {Z}^N$ such that $t_{\alpha _i}=k_i$ ($i=1,\dots ,N-1$) with the relations

$$ t_{\lambda } t_{\mu } = t_{\lambda +\mu },\quad t_{0} = 1,\quad t_{\lambda } e_{i} t_{-\lambda } = q^{(\lambda |\alpha _{i})}e_{i}, \quad t_{\lambda } f_{i} t_{-\lambda }=q^{-(\lambda |\alpha _i)}f_{i}. $$

It is a Hopf algebra with $\epsilon (t_\lambda )=1, S(t_\lambda )=t_{-\lambda },\Delta (t_\lambda )=t_\lambda \otimes t_\lambda$. We set $t_i=t_{\epsilon _i}$ so that $k_i=t_it_{i+1}^{-1}$ for $i\in \mathbb {Z}/N\mathbb {Z}$. The Hopf subalgebra $U_q{\mathfrak {gl}}_N$ is generated by $e_i,f_i$ with $1\le i\le N-1$ and all $t_i^{\pm 1}$.

The multiplicative group $\mathbb {C}^\times$ acts on $U_qL{\mathfrak {gl}}_N$ by algebra automorphisms, deforming the rescaling action of the loop parameter: the automorphism $\varphi _z$ associated with $z\in \mathbb {C}^\times$ acts as the identity on all generators except $e_0$, $f_0$, for which

$$ \varphi _z(e_0)=ze_0,\quad \varphi _z(f_0)=z^{-1}f_0. $$

The weight space of a $U_q{\mathfrak {gl}}_N$-module of weight $\mu \in \mathbb {Z}^N$ is the simultaneous eigenspace of $t_\lambda$ with eigenvalue $q^{(\mu |\lambda )}$. A $U_q{\mathfrak {gl}}_N$-module is of *type I* if it is the direct sum of its finite dimensional weight spaces. All modules considered in this paper are of type I.

### Exterior Powers of the Vector Representation

Let $V=\mathbb {C}^N$ with basis $v_1,\dots ,v_N$ and $C_N$ be the Clifford algebra of $V\oplus V^*$ with quadratic form $Q(v\oplus \alpha )=\alpha (v)$. It is the unital algebra generated by the images of the standard basis and dual basis vectors $\psi ^*_i,\psi _i$, labeled by $i\in \{1,\dots ,N\}$, which we often identify with $\mathbb {Z}/N\mathbb {Z}$, subject to the relations

1 $$\begin{aligned} \psi _{i}\psi _{j}=-\psi _{j}\psi _{i},\quad {\psi _i^*} {\psi _j^*}={-\psi _j^*}{\psi _i^*},\quad \psi _{i}{\psi _j^*}+{\psi _j^*}\psi _{i}=\delta _{ij}1. \end{aligned}$$

The Clifford algebra $C_N$ has a unique irreducible representation up to isomorphism. It is $\bigwedge V$ with $\psi ^*_i$ acting as $v\mapsto v_i\wedge v$ and $\psi _i$ by contraction. It is generated by $|0\rangle =1$ such that $\psi _i|0\rangle =0$ for all *i*. The commuting “fermion number” operators $n_i=\psi _i^*\psi _i$ have a common eigenbasis $v_{i_1}\wedge \cdots \wedge v_{i_k}$ ($i_1<\dots <i_k$) and eigenvalue 1 if $i=i_j$ for some *j* and 0 otherwise. Thus $t^{\pm 1}_i:=q^{\pm n_i}=q^{\pm 1}\psi _i^*\psi _i+\psi _i\psi _i^*$ obeys the relations

2 $$\begin{aligned} t_{i} t_i^{-1} = 1,\quad t_{i}t_{j}=t_{j} t_{i},\quad t_{i} {\psi _j^*} t_i^{-1}=q^{\delta _{ij}}{\psi _j^*},\quad t_{i}\psi _{j} t_i^{-1}=q^{-\delta _{ij}}\psi _{j}. \end{aligned}$$

for all *i*, *j*. Let $C_N(q)$ be the algebra with generators $t_i,\psi _i,\psi _i^*$, ($i=1,\dots ,N$) and relations Eqs. 1, 2. It is a $\mathbb {Z}/2\mathbb {Z}$-graded algebra with $\psi ,\psi _i^*$ odd and $t_i$ even, and $\bigwedge V$ is a $\mathbb {Z}/2\mathbb {Z}$-graded module, with the convention that $|0\rangle$ is even.

#### Proposition 2.1

(Hayashi [9]) There is a homomorphism $U_{q} L\mathfrak {gl}_{N} \rightarrow C_{N}(q)$ such that

$$ e_{i}\mapsto {\psi _i^*}\psi _{i+1},\quad f_{i}\mapsto {\psi _{i+1}^*}\psi _{i},\quad t_i^{\pm 1}\mapsto t_i^{\pm 1}, $$

for $$i\in \mathbb {Z}/N\mathbb {Z}$$.

Thus we have a representation of $U_qL{\mathfrak {gl}}_N$ on $\bigwedge V$. It is the direct sum of the irreducible eigenspaces $\bigwedge ^kV$ of the central element $\prod _{i=1}^N t_i$. These irreducible representations are generated by the highest weight vectors

3 $$\begin{aligned} v^k=\psi _k^*\cdots \psi _1^*|0\rangle \end{aligned}$$

of weight $\sum _{i=1}^k\epsilon _i$. In fact they are obtained from the representation of the classical Lie algebra $L{\mathfrak {gl}}_N$ on the same representation space and with the same action of the generators $e_i,f_i$ by setting $t_\lambda =q^{\lambda }$ for Cartan elements $\lambda$. The point is that, since the eigenvalues of $\epsilon _{i}$ are 1 or 0, the right-hand side $(q^{h_i}-q^{-h_i})/(q-q^{-1})$ of the commutation relation of $e_i,f_i$ coincides with the action of its classical counterpart $h_i=\epsilon _i-\epsilon _{i+1}$.

#### Definition 2.2

The *j-th exterior power of the vector representation* of $U_qL{\mathfrak {gl}}_N$ with spectral parameter *z* is the twist $\bigwedge ^{j} V(z)$ of $\bigwedge ^{j} V$ by the automorphism $\varphi _z$. The vector representation is $V(z)=\bigwedge ^{1} V(z)$.

Thus the action of the generators is given by the formulae of Proposition 2.1 except that $e_0,f_0$ are mapped to $z\psi _N^*\psi _{1}$ and $z^{-1}\psi _1^*\psi _N$, respectively.

#### Remark 2.3

Our definition of the exterior power representation has a slightly non-standard choice of signs. A more common convention is to let $e_0$ and $f_0$ act as $(-1)^{j-1}z\psi _N^*\psi _1$, $(-1)^{j-1}z^{-1}\psi _1^*\psi _N$, respectively, which in the limit $q\rightarrow 1$ correspond to the action of $zE_{N,1},z^{-1}E_{1,N}$, in terms of the standard basis $(E_{i,j})$ of ${\mathfrak {gl}}_N$, so that *z* has the interpretation of an “evaluation point.” Our convention is better suited to the fermionic representation and somewhat reduces the number of signs in the formulas. The relation of our definition of the exterior power $\bigwedge ^{j} V(z)$ to the definition $V^{(j)}(z)$ in [6] is $\bigwedge ^{j} V(z)=V^{(j)}((-1)^{j-1}z)$,

Another interpretation of the exterior powers, useful to compute *R*-matrices by the fusion procedure, is in terms of *q*-deformed exterior products:

#### Proposition 2.4

(Jimbo [11]) Let $k\ge 2$ and *S* be the subspace of $V^{\otimes k}$ spanned by $\alpha \otimes v_i\otimes v_i\otimes \beta$, $\alpha \otimes (v_i\otimes v_j+q v_j\otimes v_i)\otimes \beta$ for $i>j$, $\alpha \in V^{\otimes r},\beta \in V^{\otimes k-r-2}$, $r=0,\dots ,k-2$. Then *S* is a $U_{q} L {\mathfrak {gl}}_{N}$ submodule of

$$ V^k(z):=V(q^{-k+1}z)\otimes \cdots \otimes V(q^{k-3}z)\otimes V(q^{k-1}z) $$

and we have the isomorphism of $U_qL{\mathfrak {gl}}_N$-modules $V^k(z)/S\rightarrow \bigwedge ^k V(z)$ such that $v_{i_1}\otimes \cdots \otimes v_{i_k}+S\mapsto v_{i_1}\wedge \cdots \wedge v_{i_k}$ for $1\le i_1<\cdots <i_k\le N$.

### Braiding Matrices

For generic $z_1,z_2$ the tensor products module $\bigwedge ^{k} V(z_1)\otimes \bigwedge ^{k'} V(z_2)$ with action of $U_q\widehat{{\mathfrak {gl}}}_N$ given by the coproduct are irreducible and do not depend on the order of factors up to isomorphism, see [5, Proposition 12.2.15]. Thus there is an isomorphism of $U_q\widehat{{\mathfrak {gl}}}_N$-modules

$$ \check{R}_{k,k'}(z_1/z_2):\textstyle {\bigwedge ^{k} V(z_1)\otimes \bigwedge ^{k'} V(z_2)\rightarrow \bigwedge ^{k'} V(z_2)\otimes \bigwedge ^{k} V(z_1)}, $$

which, viewed as a linear map between the underlying vector spaces, depends on the ratio of spectral parameters. We find it convenient to normalize it in such a way that $v^k\otimes v^{k'}$ is mapped to $(-1)^{kk'}v^{k'}\otimes v^k$. Then the *R*-matrix

$$ R_{k,k'}(z_1/z_2)=P\check{R}_{k,k'}(z_1/z_2)\in {\text {End}}_{\mathbb {C}}(\textstyle {\bigwedge ^{k} V(z_1)\otimes \bigwedge ^{k'} V(z_2)}), $$

obtained by the composition with the graded flip $P:v\otimes w\rightarrow (-1)^{kk'}w\otimes v$, restricts to the identity on the product of highest weight spaces.

Then by the irreducibility of tensor products at generic values of the spectral parameter we have the braiding relation

4 $$\begin{aligned} (\check{R}_{k_2k_3}(z_2/z_3)&\otimes \textrm{id}) (\textrm{id}\otimes \check{R}_{k_1k_3}(z_1/z_3)) (\check{R}_{k_1k_2}(z_1/z_2)\otimes \textrm{id})\nonumber \\&= (\textrm{id}\otimes \check{R}_{k_1k_2}(z_1/z_2)) (\check{R}_{k_1k_3}(z_1/z_3)\otimes \textrm{id}) (\textrm{id}\otimes \check{R}_{k_2k_3}(z_2/z_3)), \end{aligned}$$

on $\bigwedge ^{k_1}V(z_1)\otimes \bigwedge ^{k_2}V(z_2)\otimes \bigwedge ^{k_3}V(z_3)$, equivalent to the Yang–Baxter equation for the *R*-matrices. Moreover we have the inversion (or unitarity) relation

5 $$\begin{aligned} \check{R}_{k'k}(z^{-1})\check{R}_{kk'}(z)=\textrm{id}. \end{aligned}$$

These *R*-matrices were computed by Date and Okado [6], see also [2], in terms of projections onto irreducible submodules for the action of $U_q{\mathfrak {gl}}_N$. The coefficients were computed using the fusion procedure [13]. Here we give an alternative fermionic formula.

Let $\bigwedge V\otimes \bigwedge V$ be the tensor product of $\mathbb {Z}/2\mathbb {Z}$-graded vector spaces. The action of tensor products of linear maps is given by the sign rule: $(f\otimes g)(v\otimes w)=(-1)^{|g||v|}fv\otimes gw$ for *g* of degree |*g*| and *v* of degree |*v*|. We introduce the following even endomorphisms of $\bigwedge V\otimes \bigwedge V$:

6 $$\begin{aligned} E=\sum _{i=1}^{N} (\psi ^{*}_i\otimes {\psi }_i)\prod _{j>i}{K}_{j} \qquad F=-\sum _{i=1}^{N} \prod _{j<i}{K}^{{-1}}_j({\psi }_i\otimes \psi ^{*}_i) \qquad {K}=\prod _{i=1}^N{{K}}_i, \end{aligned}$$

with $$K_i=t_i\otimes t_i^{-1}$$ ($$i=1,\dots ,N$$).

#### Lemma 2.5

The endomorphisms *E*, *F*, *K* commute with the action of $U_q{\mathfrak {gl}}_N$ and obey the commutation relations of $U_q\mathfrak {sl}_2$:

$$ [E,F]=\frac{K-K^{-1}}{q-q^{-1}},\quad KEK^{-1}=q^2E,\quad KFK^{-1}=q^{-2}F. $$

This is an instance of Howe duality and is discussed in the next section. It is convenient to extend this action to an action of $U_q{\mathfrak {gl}}_2$ by setting $T_1=\prod _{i=1}^N t_i\otimes 1$, $T_2=\prod _{i=1}^N1\otimes t_i$ so that $\bigwedge ^{k}V\otimes \bigwedge ^{k'}V$ is the weight space of weight $(k,k')$.

#### Theorem 2.6

The braiding matrix

$$ \check{R}_{k,k'}(z_1/z_2):\textstyle {\bigwedge ^kV(z_1)\otimes \bigwedge ^{k'}V(z_2)\rightarrow \bigwedge ^{k'}V(z_2)\otimes \bigwedge ^{k}V(z_1)}, $$

normalized so that $\check{R}_{k,k'}(z_1/z_2)\,v^k\otimes v^{k'}=(-1)^{kk'}v^{k'}\otimes v^{k}$, is given by

$$ \check{R}_{k,k'}(z_1/z_2)=(-1)^{\min (k,k')}A_{k-k'}(z_1/z_2) $$

where $A_{k-k'}(z_1/z_2)$ is the series in $U_q\mathfrak {gl}_2$ defined by

$$ A_m(z)=\sum _{j=0}^\infty (-q)^{j}\frac{1-q^{|m|}z}{1-q^{2j+|m|}z} {\left\{ \begin{array}{ll} E^{(j)}F^{(j+m)},&  \text {if } m \ge 0, \\ E^{(j-m)}F^{(j)},&  \text {if } m \le 0, \end{array}\right. } $$

with the divided power notation $E^{(l)}=E^l/[l]_q!$, $F^{(l)}=F^l/[l]_q!$. In the sum over *j* only the terms with *j* in the range

$$ 0\le j \le \min (k,k',N-k,N-k'). $$

contribute non-trivially.

The proof relies on Howe duality, which we explain in the next section, and the computation [6] of the scalar by which $\check{R}$ acts on each irreducible subrepresentation of the tensor product.

### Limit $N\rightarrow \infty$ and Braiding Matrices on Fock Spaces

Our formula for braiding matrices on exterior powers of vector representations does not depend explicitly on the dimension *N* and have a formal limit as $N\rightarrow \infty$ to braiding matrices acting on Fock spaces realized following Dirac as semi-infinite forms $\mathcal {F}=\oplus _k\bigwedge ^{\frac{\infty }{2}+k}\mathbb {C}^{\infty }$. Here we show that the limit can be rigorously constructed so that our braiding matrices converge to braiding matrices defined on Fock spaces.

Let $C_\infty$ be the infinite dimensional Clifford algebra generated by $b_n$, $c_n$, $n\in \mathbb {Z}+\frac{1}{2}$, with relations $b_nc_m+c_mb_n=\delta _{n,-m}$. The Fock space $\mathcal {F}$ is the module over $C_\infty$ generated by a vacuum vector $|\textrm{vac}\rangle$ annihilated by $b_n$ and $c_n$ for $n>0$ and carries a representation of Lie algebras of infinite matrices, see e.g. [12], Lecture 4. The Clifford algebra has a $\mathbb {Z}$-grading so that $b_n$ has degree 1 and $c_n$ has degree $-1$ for all *n*. We have a compatible grading

$$ \mathcal {F}=\oplus _{k\in \mathbb {Z}}\mathcal {F}_k. $$

defined by giving degree 0 to $|\textrm{vac}\rangle$.

We realize $\mathcal {F}_k$ as a direct limit of $\mathcal {F}_k^{(N)}=\bigwedge ^{N/2+k}\mathbb {C}^N$ as $N\rightarrow \infty$ with *N* even.

Namely we renumber the standard basis of $\mathbb {C}^N$ as $v_{-\frac{N-1}{2}},\dots ,v_{\frac{N-3}{2}},v_{\frac{N-1}{2}}$ to identify $\mathbb {C}^N$ as a subspace of $\mathbb {C}^{N+2}$ for each *N*, and we embed $i_N:\bigwedge \mathbb {C}^N\hookrightarrow \bigwedge \mathbb {C}^{N+2}$ as

$$ i_N(\alpha )=\alpha \wedge v_{\frac{N+1}{2}},\quad N\in 2\mathbb {N}. $$

Then $i_N$ is a homomorphism of modules over the subalgebra $C_{N}$ generated by $b_n,c_n$ for $|n|\le (N-1)/2$, so that $b_n$ is the exterior product with $v_n$ and $c_{-n}$ is the contraction with the dual $v_n^*$. It is a *graded* homomorphism if we assign degree *k* to the subspace $\mathcal {F}_k^{(N)}=\bigwedge ^{\frac{N}{2}+k}\mathbb {C}^N$. The relation to the operators on $\bigwedge \mathbb {C}^N$ of Section 2.3 is

$$ \psi ^*_n=b_{-\frac{N+1}{2}+n},\quad \psi _n=c_{\frac{N+1}{2}-n},\quad (n=1,\dots ,N) . $$

Let

$$ |\textrm{vac}\rangle ^{(N)}=v_{1/2}\wedge v_{3/2}\wedge \cdots \wedge v_{\frac{N-1}{2}}. $$

Then $b_n|\textrm{vac}\rangle ^{(N)}=0=c_n|\textrm{vac}\rangle ^{(N)}$ for $n>0$ and $i_N$ maps $|\textrm{vac}\rangle ^{(N)}$ to $|\textrm{vac}\rangle ^{(N+2)}$. Thus $\mathcal {F}^{(N)}=\bigwedge \mathbb {C}^N$ is identified with the subspace of $\mathcal {F}$ generated by $|\textrm{vac}\rangle$ over $C_N$ and we have a filtration of graded vector spaces

$$ \cdots \subset \mathcal {F}^{(N)}\subset \mathcal {F}^{(N+2)}\subset \cdots ,\quad N\in 2\mathbb {Z}, $$

with direct limit $\cup _{N}\mathcal {F}^{(N)}=\mathcal {F}$. In particular,

$$ \mathcal {F}_k=\cup _N\mathcal {F}_k^{(N)} $$

The renormalized fermion number $\tilde{n}_i\in C_N$ is

$$ \tilde{n}_i=\,:b_ic_{-i}:= {\left\{ \begin{array}{ll} b_ic_{-i}&  \text {if } i<0, \\ -c_{-i}b_i&  \text {if } i>0. \end{array}\right. } $$

It commutes with $i_N$ and is normalized so that $\tilde{n}_i|\textrm{vac}\rangle ^{(N)}=0$. Then $\tilde{t}_i=q^{\tilde{n}_i}$ is well-defined on the direct limit. It has the properties

7 $$\begin{aligned} \tilde{t}_n|\textrm{vac}\rangle =1,\quad \tilde{t}_nb_m\tilde{t}_n^{-1}=q^{\delta _{n,m}}b_m,\quad \tilde{t}_nc_{-m}\tilde{t}_n^{-1}=q^{-\delta _{n,-m}}c_{-m}, \end{aligned}$$

for all $n,m\in \mathbb {Z}+\frac{1}{2}$. The restriction of $\tilde{t}_n$ to $\mathcal {F}^{(N)}$ is related to the generator $t_n$ of Section 2.3 by $\tilde{t}_n=q^{-N/2}t_{n+(N-1)/2}$.

The representation of $U_q\mathfrak {sl}_2$ on $\mathcal {F}^{(N)}\otimes \mathcal {F}^{(N)}$ obtained by Howe duality defined by Eq. 6 is then given in this new notation by the following action of generators (where we add the dependence on *N* in the notation):

$$\begin{aligned} E(N)&=\sum _{i=-\frac{N-1}{2}}^{\frac{N-1}{2}} (b_i\otimes c_{-i})\prod _{j=i+1}^{\frac{N-1}{2}}\tilde{K}_j, \quad F(N)=-\sum _{i=-\frac{N-1}{2}}^{\frac{N-1}{2}} \prod _{j=-\frac{N-1}{2}}^{i-1}\tilde{K}_j(c_{-i}\otimes b_i) \\ K(N)&=\prod _{i=-\frac{N-1}{2}}^{\frac{N-1}{2}}\tilde{K}_i,\quad \tilde{K}_i=\tilde{t}_i\otimes \tilde{t}_i^{-1} \end{aligned}$$

#### Proposition 2.7

Let $X(N)=E(N),F(N),$ or *K*(*N*) and $N\in 2\mathbb {Z}$. Then

$$ (i_N\otimes i_N)\circ X(N)=X(N+2)\circ (i_N\otimes i_N). $$

Thus *E*(*N*), *F*(*N*), *K*(*N*) are the restrictions to the invariant subspace $\mathcal {F}^{(N)}\otimes \mathcal {F}^{(N)}\subset \mathcal {F}\otimes \mathcal {F}$ of well-defined operators $E,F,K\in {\text {End}}(\mathcal {F}\otimes \mathcal {F})$ defining a representation of $U_q\mathfrak {sl}_2$.

#### Proof

Since $\mathcal {F}^{(N)}$ is generated by $|\textrm{vac}\rangle ^{(N)}$ as a module over the Clifford algebra $C_N$, $\tilde{n}_i$ is uniquely characterized by the commutation relations (7) and the condition that $\tilde{n}_i|\textrm{vac}\rangle ^{(N)}=0$. Thus for $N<N'$ and $|i|>(N-1)/2$, $\tilde{t}_i$ acts as 1 on $\mathcal {F}^{(N)}\subset \mathcal {F}^{(N')}$. In particular for $|i|=(N+1)/2$, $\tilde{K}_i$ acts by 1 on the image of $\mathcal {F}^{(N)}$ in $\mathcal {F}^{(N+2)}$ and thus $K(N+2)\circ (i_N\otimes i_N)=(i_N\otimes i_N)\circ K(N)$. As for *E*(*N*), we have similarly

$$ (i_N\otimes i_N)\circ \prod _{j=i+1}^{\frac{N-1}{2}}\tilde{K}_j=\prod _{j=i+1}^{\frac{N+1}{2}}\tilde{K}_j\circ (i_N\otimes i_N), $$

since the last factor on the right-hand side acts by 1. Also $b_i\otimes c_{-i}$ commutes with $i_N\otimes i_N$ for $|i|\le \frac{N-1}{2}$ and acts by zero on the image of $i_N\otimes i_N$ if $|i|=(N+1)/2$. This is because for $|i|>(N-1)/2$, $b_i$ and $c_{-i}$ commute (up to sign) with $C_N$ and either $c_{-i}|\textrm{vac}\rangle ^{({N+2})}$ or $b_i|\textrm{vac}\rangle ^{({N+2})}$ vanishes. Thus $E(N+2)\circ i_N\otimes i_N=i_N\otimes i_N\circ E(N)$ and similarly for *F*(*N*). $\square$

We can formally write the limiting representation of $U_q\mathfrak {sl}_2$ on $\mathcal {F}\otimes \mathcal {F}$ as

$$\begin{aligned} E&=\sum _{i=-\infty }^{\infty } (b_i\otimes c_{-i})\prod _{j=i+1}^{\infty }\tilde{K}_j, \quad F=-\sum _{i=-\infty }^{\infty } \prod _{j=-\infty }^{i-1}\tilde{K}_j(c_{-i}\otimes b_i) \\ K&=\prod _{i=-\infty }^{\infty }\tilde{K}_i,\quad \tilde{K}_i=\tilde{t}_i\otimes \tilde{t}_i^{-1} \end{aligned}$$

with the remark that, when acting with these operators on any element of $\mathcal {F}\otimes \mathcal {F}$, all but finitely many summands in the infinite sums vanish and all but finitely many factors in the products are equal to 1.

#### Theorem 2.8

Let $E^{(j)}=E/[j]_q!$, $F^{(j)}=F/[j]_q!$, $(j=0,1,\dots )$. For every $m\in \mathbb {Z}$ the operator

$$ A_m(z)=\sum _{j=0}^\infty (-q)^{j}\frac{1-q^{|m|}z}{1-q^{2j+|m|}z} {\left\{ \begin{array}{ll} E^{(j)}F^{(j+m)},&  \text {if } m\ge 0,\\ E^{(j-m)}F^{(j)},&  \text {if } m\le 0, \end{array}\right. } $$

is a well-defined rational function with values in ${\text {End}}(\mathcal {F} \otimes \mathcal {F})$. The braiding matrix

$$ \check{R}_{k,k'}(z)= (-1)^{\min (k,k')}A_{k-k'}(z) $$

maps $\mathcal {F}_k\otimes \mathcal {F}_{k'}$ to $\mathcal {F}_{k'}\otimes \mathcal {F}_k$ and is a solution of the Yang–Baxter equation (4) and the inversion relation (5).

#### Proof

The operators *E* and *F* preserve the finite dimensional subspaces $\mathcal {F}^{(N)}\otimes \mathcal {F}^{(N)}$ and they are nilpotent there so that the sums defining $A_m$ reduce to finite sums. Moreover $\check{R}_{k,k'}(z)$ restricts to the braiding matrix of Theorem 2.6 on these subspaces, so the Yang–Baxter equation and the inversion relation follow. $\square$

## Quantum Skew Howe Duality

The classical skew-symmetric version of Howe duality [10, Section 4] states that the natural actions of $$ GL _N$$ and $$ GL _M$$ on $$\bigwedge (\mathbb {C}^N\otimes \mathbb {C}^M)$$ generate each other’s commutant and one has a decomposition into irreducibles of $$ GL _N\times GL _M$$

$$ \bigwedge (\mathbb {C}^N\otimes \mathbb {C}^M)\cong \bigoplus _{\lambda }V^N_\lambda \otimes V^M_{\lambda ^t}. $$

The sum is over all Young diagrams $$\lambda $$ with at most *N* rows such that the transposed diagrams $$\lambda ^t$$ has at most *M* rows (equivalently, the length of the first row of $$\lambda $$ is at most *M*) and $$V^N_\lambda $$ denotes the irreducible representation of $$ GL _N$$ of highest weight $$\lambda $$. The following nice pictorial description of the corresponding highest weight vectors is due to Howe in *loc. cit.*: write the tensor products of basis vectors $$v_{i,r}=v_i\otimes w_r$$ $$(i=1,\dots ,N, r=1,\dots , M)$$ in the boxes of an $$N\times M$$ grid (the tensors $$v_{1,r}$$ go in the first row, $$v_{2,r}$$ in the second row, etc.). The Young diagrams occuring in the decomposition fit in this rectangle. The highest weight vector corresponding to $$\lambda $$ is obtained by taking the wedge product of the basis vectors in the boxes of the Young diagram $$\lambda $$. For example, if $$\lambda =(3,1)$$, we take $$v_{1,1}\wedge v_{1,2}\wedge v_{1,3}\wedge v_{2,1}$$. It is indeed of weight $$\lambda $$ for $$ GL _N$$ and of weight $$\lambda ^t$$ for $$ GL _M$$.

The quantum group version of Howe duality is known: in the analogous case of the symmetric algebra it is discussed in [1, 27] and [29]. The skew-symmetric case we use here is explained in [3] (for $M=2$), [14] and [4]. Here we present an equivalent variant based on the Clifford algebra action.

Let $V=\mathbb {C}^N$ with basis $v_1,\dots ,v_N$ be the vector representation of $U_q\mathfrak {gl}_N$ and $W=\mathbb {C}^M$ with basis $w_1,\dots ,w_M$ be the vector representation of $U_q\mathfrak {gl}_M$. Then we have an isomorphism of graded commutative algebras

$$ \bigwedge (V\otimes W)\cong \bigwedge (V\oplus \cdots \oplus V) \rightarrow \left( \bigwedge V\right) ^{\otimes M} =\bigwedge V\otimes \cdots \otimes \bigwedge V $$

sending $v_i\otimes w_j$ to $1\otimes \cdots \otimes 1\otimes v_i\otimes 1\otimes \cdots \otimes 1$ with $v_i$ placed in the *j*-th factor. The right-hand side is a tensor product of $U_q\mathfrak {gl}_N$-modules and thus $U_q\mathfrak {gl}_N$ acts on it by the iterated coproduct. Similarly we have an isomorphism $\bigwedge (V\otimes W)\cong \bigwedge W\otimes \cdots \otimes \bigwedge W$ sending $v_i\otimes w_j$ to $1\otimes \cdots \otimes 1\otimes w_j\otimes 1\otimes \cdots \otimes 1$ with *i*-th factor $w_j$. Here we let $U_q\mathfrak {gl}_M$ act via the *opposite* coproduct $\Delta '=\sigma \circ \Delta$, with $\sigma (a\otimes b)=b\otimes a$.

### Proposition 3.1

The actions of $(U_q\mathfrak {gl}_N,\Delta )$ and $(U_q\mathfrak {gl}_M,\Delta ')$ on $\bigwedge (V\otimes W)$ pulled back by the isomorphisms

$$ \left( \bigwedge W\right) ^{\otimes N}\leftarrow \bigwedge (V\otimes W) \rightarrow \left( \bigwedge V\right) ^{\otimes M} $$

commute. If *q* is not a root of unity the images of $U_q\mathfrak {gl}_N$ and $U_q\mathfrak {gl}_M$ in ${\text {End}}\bigwedge (V\otimes W)$ are commutants of each other and we have a decomposition

8 $$\begin{aligned} \bigwedge (V\otimes W)\cong \bigoplus _\lambda V^N_\lambda \otimes V^M_{\lambda ^t} \end{aligned}$$

into simple $U_q\mathfrak {gl}_N\otimes U_q\mathfrak {gl}_M$-modules, where $\lambda$ runs over Young diagrams with at most *N* rows and at most *M* columns.

For the proof we view $\bigwedge (V\otimes W)$ as a representation of the Clifford algebra of $V\otimes W\oplus (V\otimes W)^*$. This algebra has generators $\psi ^*_{i,r},\psi _{i,r}$ corresponding to the tensor basis $v_i\otimes w_r$ and its dual basis. As in Section 2.3 we introduce the operators $t_{i,r}^{\pm 1}=q^{\pm 1}\psi _{i,r}^*\psi _{i,r}+\psi _{i,r}\psi _{i,r}^*$. Via the isomorphism to $(\bigwedge V)^{\otimes M}$, $\psi _{i,r}^*$ acts as $\textrm{id}^{\otimes (r-1)}\otimes \psi _i^*\otimes \textrm{id}^{\otimes (N-r)}$ and $\psi _{i,r}$ acts as $\textrm{id}^{\otimes (r-1)}\otimes \psi _i\otimes \textrm{id}^{\otimes (N-r)}$. Thus the pull-back of the action of $U_q\mathfrak {gl}_N$ on $\bigwedge (V\otimes W)$ is given on generators by

$$\begin{aligned} e_i&\mapsto \sum _{r=1}^M\prod _{s=1}^{r-1} k_{i,s} \psi ^*_{i,r}\psi _{i+1,r}, \quad k_{i,s}=t_{i,s}t_{i+1,s}^{-1}\\ f_i&\mapsto \sum _{r=1}^M \psi ^*_{i+1,r}\psi _{i,r}\prod _{s={r+1}}^M k_{i,s}^{-1} \\ t_i^{\pm 1}&\mapsto \prod _{r=1}^Mt_{i,r}^{\pm 1}. \end{aligned}$$

Similary we can pull-back the action of $U_q\mathfrak {gl}_M$ on $(\bigwedge W)^{\otimes N}$ with the opposite coproduct. The action of generators (denoted by capital letters to distinguish them)

$$\begin{aligned} E_r&\mapsto \sum _{i=1}^N\psi ^*_{i,r}\psi _{i,r+1} \prod _{j=i+1}^{N} K_{j,r},\quad K_{j,r}=t_{j,r}t_{j,r+1}^{-1}, \\ F_r&\mapsto \sum _{i=1}^N \prod _{j=1}^{i-1} K_{j,r}^{-1}\psi ^*_{i,r+1}\psi _{i,r}, \\ T_r^{\pm 1}&\mapsto \prod _{i=1}^Nt_{i,r}^{\pm 1}. \end{aligned}$$

In the case $M=2$ this reduces to the formulae of the previous section, by remembering the sign rule in the definition of *F*. By construction, we obtain actions of $U_q{\mathfrak {gl}}_N$ and $U_q{\mathfrak {gl}}_M$ and we need to check that they commute:

### Lemma 3.2

The actions of $U_q{\mathfrak {gl}}_N$ and $U_q{\mathfrak {gl}}_M$ on $\bigwedge (V\otimes W)$ commute.

### Proof

It is clear that $t_i^{\pm 1}$ commutes with the action of $U_q{\mathfrak {gl}}_M$ and that $T_r^{\pm 1}$ commutes with the action of $U_q{\mathfrak {gl}}_N$. Let us check that $[e_i,E_r]=0$. The cases of the other generators are dealt with in the same way. The only terms in the sum defining $e_i$ and $E_r$ that contribute non-trivially to the commutator are

$$\begin{aligned} [e_i,E_r]&=\prod _{s<r}k_{i,s} [\psi _{i,r}^*\psi _{i+1,r}+k_{i,r}\psi _{i,r+1}^*\psi _{i+1,r+1}, \\&\quad \psi _{i,r}^*\psi _{i,r+1}K_{i+1,r}+ \psi _{i+1,r}^*\psi _{i+1,r+1}] \prod _{j>i+1}K_{j,r}. \end{aligned}$$

The bracket on the right-hand side is

$$\begin{aligned}&[\psi _{i,r}^*\psi _{i+1,r}, \psi _{i+1,r}^*\psi _{i+1,r+1}] +[k_{i,r}\psi _{i,r+1}^*\psi _{i+1,r+1}, \psi _{i,r}^*\psi _{i,r+1}K_{i+1,r}] \\&=\psi _{i,r}^*\psi _{i+1,r+1} +k_{i,r}\psi _{i+1,r+1}\psi _{i,r}^*K_{i+1,r} \\&=\psi _{i,r}^*\psi _{i+1,r+1}+t_{i,r}\psi _{i+1,r+1}\psi _{i,r}^*t_{i+1,r+1}^{-1} \end{aligned}$$

We have the relations $t_{i,r}\psi _{i,r}^*=q\psi _{i,r}^*$ and $\psi _{i+1,r+1}t_{i+1,r+1}^{-1}=q^{-1}\psi _{i+1,r+1}$ and we are left with $\psi _{i,r}^*\psi _{i+1,r+1}+\psi _{i+1,r+1}\psi _{i,r}^*=0$. $\square$

To complete the proof of Proposition 3.1, we observe that for *q* not a root of unity the decomposition into irreducible representations in the classical case deforms to the quantum group case. In fact the highest weight vectors generating irreducible $U_q{\mathfrak {gl}}_N\otimes U_q{\mathfrak {gl}}_M$-modules are still given by Howe’s construction.

### Proof of Theorem 2.6

We use the formula of Date and Okado [6] for the action of the *R*-matrix on highest weight vectors of irreducible $U_q{\mathfrak {gl}}_N$-subrepresentations in $\bigwedge ^kV\otimes \bigwedge ^{k'}V$. The decomposition into irreducibles is best understood in terms of Howe duality: we consider the case $M=2$ of the decomposition (8) of Proposition 3.1. The left-hand side is

$$ \textstyle {\bigwedge (V\otimes \mathbb {C}^2)=\bigwedge (V\oplus V)= \bigwedge V\otimes \bigwedge V=\oplus _{k,k'=0}^N\bigwedge ^kV\otimes \bigwedge ^{k'} V} $$

and the summand labeled by $(k,k')\in \{0,\dots ,N\}^2$ is the weight space of weight $(k,k')$ for the $U_q{\mathfrak {gl}}_2$-action. The right-hand side is a sum over Young diagram $\lambda$ with at most two columns and at most *N* rows. Thus $\lambda =(2,\dots ,2,1,\dots ,1)=2^{\ell '}1^{\ell -\ell '}$ ($\ell '$ rows of length 2, $\ell -\ell '$ rows of length 1) and $\lambda ^t=(\ell ,\ell ')$ with $N\ge \ell \ge \ell '\ge 0$. The irreducible $U_q{\mathfrak {gl}}_2$-module $V^2_{(\ell ,\ell ')}$ has a non-trivial weight space of weight $(k,k')$ if and only if $k+k'=\ell +\ell '$ for some $s\ge 0$ and $\ell '-\ell \le k-k'\le \ell -\ell '$. In this case the weight space is one-dimensional. If $k\ge k'$ the corresponding highest weights are

$$ (\ell ,\ell ')=(k,k'),(k+1,k'-1),\dots ,(k+n,k'-n), $$

where $n=\min (k',N-k)$, whereas if $k\le k'$ they are

$$ (\ell ,\ell ')=(k',k),(k'+1,k-1),\ldots ,(k'+n,k-n), $$

where $n=\min (k,N-k')$. In both cases we can write

$$ n=\min (k,k',N-k,N-k'). $$

With this notation, the weight $(k,k')$ subspace in the decomposition (8) gives the decomposition

$$ {\textstyle \bigwedge ^{k}V\otimes \bigwedge ^{k'}V}\cong \oplus _{s=0}^{n} V^N_{(\overline{k}+s,\underline{k}-s)^t},\quad \text {with } \overline{k}=\max (k,k') \text { and } \underline{k}=\min (k,k'), $$

as an $U_q{\mathfrak {gl}}_N$-module. The $s=0$ component is generated by the highest weight vector $v^{(\overline{k},\underline{k})}_0=v^{\overline{k}} \otimes v^{\underline{k}}$, see Eq. 3, and the highest weight vector $v^{(k,k')}_s$ of $V^N_{(\overline{k}+s,\underline{k}-s)^t}$ is proportional to $F^{(s)}v_0^{(k+s,k'-s)}$ if $k\ge k'$ and to $F^{(k'-k+s)}v_0^{(k'+s,k-s)}$ if $k\le k'$. We normalize it as in [6] by fixing the coefficient of the basis vector $\psi ^*_k\cdots \psi ^*_1|0\rangle$ in the first factor. Namely,

$$ v_s^{(k,k')}=\psi _k^*\cdots \psi _1^*|0\rangle \otimes \psi ^*_{\overline{k}+s}\cdots \psi ^*_{\overline{k}+1}\psi ^*_{\underline{k}-s}\cdots \psi _1^*|0\rangle +\cdots . $$

Let

$$ \textstyle Q_s:\bigwedge ^k V\otimes \bigwedge ^{k'}V\rightarrow \bigwedge ^{k'} V\otimes \bigwedge ^{k}V $$

be the unique $U_q{\mathfrak {gl}}_N$-linear map sending $v^{(k,k')}_s$ to the vector of the same weight $v^{(k',k)}_{s'}$, $s'=k-k'+s$ and vanishing on the other irreducible components. In our notation and with the normalization of Theorem 2.6, the formula of [6], obtained by the fusion procedure, is

9 $$\begin{aligned} \check{R}_{k,k'}(z) =\sum _{s=0}^{n}(-1)^{kk'+(k-k')s}\prod _{j=1}^s\frac{z-q^{|k-k'|+2j}}{1-z q^{|k-k'|+2j}}Q_s. \end{aligned}$$

To deduce a formula in terms of the $U_q{\mathfrak {gl}}_2$-action we need to compare the normalizations of the highest weight vectors. If $k\ge k'$ a straightforward computation using the formula for *F* in Eq. 6 shows that

$$ v^{(k,k')}_s=(-1)^{sk}q^{ss'}F^{(s)}v^{(k+s,k'-s)}_0, \quad v^{(k',k)}_{s'}=(-1)^{s'k'}q^{ss'}F^{(s')}v^{(k+s,k'-s)}_0. $$

It follows that for $k\ge k'$

$$ \check{R}_{k,k'}(z)F^{(s)}v_0^{(k+s,k'-s)} =(-1)^{k'}\prod _{j=1}^s\frac{z-q^{|k-k'|+2j}}{1-z q^{|k-k'|+2j}}F^{(s')}v_0^{(k+s,k'-s)} $$

To prove the formula for $\check{R}$ given in the Theorem we need to compute the action of $A_{k-k'}(z)$ on the irreducible representation generated by the highest weight vector $v^{(k+s,k'-s)}_0$ of $\mathfrak {sl}_2$-weight $\ell =k-k'+2s\in \mathbb {Z}_{\ge 0}$. Comparing with the formula for $A_m(z)$ from Appendix A (see Lemma A.1) we see that $\check{R}_{k,k'}(z)$ acts by the same factor on $F^{(s)}v_0^{(k+s,k'-s)}$ up to the sign $(-1)^{k'}=(-1)^{\min (k,k')}$.

If $k\le k'$ the reasoning is similar: in this case the highest weight vector $v^{(k,k')}_s$ is proportional to $F^{(s')}v_0^{(k'+s,k-s)}$ with $s'=k'-k+s$ and

$$ v^{(k,k')}_s=(-1)^{sk'}q^{ss'}F^{(s')}v^{(k'+s,k-s)}_0, \quad v^{(k',k)}_{s'}=(-1)^{s'k}q^{ss'}F^{(s)}v^{(k'+s,k-s)}_0. $$

From this we obtain

$$ \check{R}_{k,k'}(z)F^{(s')}v_0^{(k'+s,k-s)} =(-1)^{k}\prod _{j=1}^s\frac{z-q^{|k-k'|+2j}}{1-z q^{|k-k'|+2j}}F^{(s)}v_0^{(k'+s,k-s)}, $$

which is the same as the action of $A_{k-k'}(z)$ up to the sign $(-1)^{k}=(-1)^{\min (k,k')}$. $\square$

## Dynamical Weyl Group

### Braiding Matrices as Generators of the Weyl Group

In Theorem 2.6 the braiding matrix for the tensor product of two representations of $U_qL\mathfrak {gl}_N$ obtained from exterior powers of the vector representation of $U_q\mathfrak {gl}_N$ are expressed in terms of the action of $U_q\mathfrak {gl}_2$ via Howe duality. In this section we consider braiding matrices on the tensor product of *M* exterior powers and notice that they are correspondingly expressed in terms of the action of $U_q\mathfrak {gl}_M$ via the simple root embeddings of $U_q{\mathfrak {gl}}_2$. The braid and inversion relations of the $\check{R}$-matrices acting on a *M*-fold tensor product is then translated by Howe duality to braid and inversion relations in $U_q{\mathfrak {gl}}_M$. The result is then that these are relations of the dynamical Weyl group.

Recall that the (ordinary) Weyl group of $$ GL _M$$ is the symmetric group $$S_M$$. It acts on $$\mathbb {C}^M$$ and the weight lattice $$\mathbb {Z}^M$$ by permutations. It is generated by the transpositions $$s_i\in S_M$$ of *i* and $$i+1$$ ($$i=1,\dots ,M-1$$) with the relations $$s_i^2=1$$, $$s_is_j=s_js_i$$ for $$|i-j|\ge 2$$ and $$s_is_{i+1}s_i=s_{i+1}s_is_{i+1}$$ for $$1\le i\le M-2$$; the dynamical Weyl group relations are a version with parameters.

Let us define formal series $A_{i,\mu }(z)$ of rational functions of *M*-variables $z=(z_1,\dots ,z_M)$ with values in $U_q{\mathfrak {sl}}_M\subset U_q{\mathfrak {gl}}_M$ for $1\le i\le M-1$ and $\mu \in P_M=\mathbb {Z}^M$:

$$ A_{i,\mu }(z)=\sum _{j=0}^{\infty } (-q)^{j} \frac{z_{i+1}-q^{|\mu _i-\mu _{i+1}|}z_i}{z_{i+1}-q^{2j+|\mu _i-\mu _{i+1}|}z_i} E_i^{(j+(\mu _{i+1}-\mu _i)_+)}F_i^{(j+(\mu _i-\mu _{i+1})_+)}. $$

Here we set $(x)_+=\max (x,0)$ and, as before, we denote the generators by capital letters to distinguish them from the generators of $U_q{\mathfrak {gl}}_N$. For any finite dimensional $U_q{\mathfrak {gl}}_N$-module *U* of type I only finitely many terms contribute non-trivially to the sum and $A_{i,\mu }(z)$ maps the weight space $U[\mu ]$ of weight $\mu$ to $U[s_i\mu ]$. Notice that $A_{i,\mu }(z)$ depends on $\mu$ through its class in the weight lattice $P_M=\mathbb {Z}^M/\mathbb {Z}(1,\dots ,1)$ of $\mathfrak {sl}_M$. For $M=2$, the map $\mathbb {Z}^2\rightarrow \mathbb {Z}$, $\mu \mapsto \mu _1-\mu _2$ induces a bijection $P_2\cong \mathbb {Z}$. With this identification, $A_{1,m}(z_1,z_2)=A_m(z_1/z_2)$ where

10 $$\begin{aligned} A_m(z)= \sum _{j\ge 0} (-q)^{j}\frac{1-q^{|m|}z}{1-q^{2j+|m|}z} {\left\{ \begin{array}{ll} E^{(j)}F^{(j+m)},&  \text {if } m\ge 0, \\ E^{(j+|m|)}F^{(j)},&  \text {if } m\le 0. \end{array}\right. } \end{aligned}$$

The general series $A_{i,\mu }(z)$ reduces to this case via the embedding $j_i:U_q\mathfrak {sl}_2\hookrightarrow U_q\mathfrak {sl}_M$ sending $E,F,K^{\pm 1}$ to $E_i,F_i,K_i^{\pm 1}$:

$$ A_{i,\mu }(z)=j_i\left( A_{\mu (h_i)}(z_i/z_{i+1})\right) . $$

By construction, see Theorem 2.6, the action of $A_{i,\mu }(z)$ on the tensor product of exterior powers $\bigwedge ^{\mu _i}V(z_i)$ via Howe duality is the action of the braiding matrix $\check{R}_{\mu _i,\mu _{i+1}}(z_i/z_{i+1})$ on the *i*th and $i+1$st factors (up to an irrelevant sign). Note that this tensor product is the weight space of weight $\mu$ for the action of $U_q{\mathfrak {gl}}_M$ on the tensor product of *M* copies of $\bigwedge V$.

#### Theorem 4.1

Let *U* be any finite dimensional $U_q{\mathfrak {gl}}_M$-module of type I. Then the restriction of $A_{i,\mu }(z)$ to the weight space $U[\mu ]$ of weight $\mu$ obeys the following relations.

(i): For $1\le i,j\le M-1$ such that $|i-j|\ge 2$,
  $$ A_{i,s_i\mu }(s_jz)A_{j,\mu }(z) = A_{j,s_j\mu }(s_iz)A_{i,\mu }(z), $$
  in $${\text {Hom}}(U[\mu ],U[s_is_j\mu ])$$.
(ii): For all $1\le i\le M-1$,
  $$ A_{i,s_i\mu }(s_iz)A_{i,\mu }(z) =\textrm{id} $$
  in $${\text {End}}(U[\mu ])$$.
(iii): For $$1\le i\le M-2$$,
  $$\begin{aligned} A_{i,s_{i+1}s_i\mu }&(s_{i+1}s_iz) A_{i+1,s_i\mu }(s_iz)A_{i,\mu }(z) \\&= A_{i+1,s_is_{i+1}\mu }(s_is_{i+1}z) A_{i,s_{i+1}\mu }(s_{i+1}z)A_{i+1,\mu }(z), \end{aligned}$$
  in $${\text {Hom}}(U[\mu ],U[s_is_{i+1}s_i\mu ])$$.

#### Proof

By complete reducibility we may assume that *U* is irreducible. The relations hold for the action of $A_{i,\mu }$ on $\otimes _{i=1}^M\bigwedge ^{\mu _i}V(z_i)$ via Howe duality: (i) follows from the fact that the corresponding $\check{R}$-matrices act on different factors of the tensor product, (ii) is the inversion relation (5) and (iii) is the braiding relation (4). Thus the relations hold for any irreducible $U_q{\mathfrak {gl}}_M$-module occurring in the decomposition of the tensor product. But from Proposition 3.1 we see that any partition does occurs as a highest weight if we take *N* sufficiently large. This covers all type I irreducible representations of $U_q\mathfrak {sl}_M$. For $U_q{\mathfrak {gl}}_M$ we can obtain all representations by tensoring these with a power of the one dimensional representations on which $U_q\mathfrak {sl}_M$ acts trivially (i.e. by the counit) and $t_i$ act by $q^{-1}$ for all *i*. This has the effect of shifting the $\mu _i$s by a common amount and does not affect the validity of the claim, since $A_{i,\mu }(z)$ depends on differences of $\mu _i$s. $\square$

This result can be reformulated as the construction of a representation of the Weyl group on *U*-valued functions. Let *U* be a finite-dimensional type I $U_q{\mathfrak {gl}}_M$-module with weight decomposition $U=\oplus _\mu U[\mu ]$. Let $A_{i,U}(z)$ be the ${\text {End}}U$-valued rational function such that

11 $$\begin{aligned} A_{i,U}(z)|_{U[\mu ]}=A_{i,\mu }(z):U[\mu ]\rightarrow U[s_i\mu ] \end{aligned}$$

for all weights $\mu$.

#### Corollary 4.2

Let *U* be a finite-dimensional $U_q{\mathfrak {gl}}_M$-module of type I. The endomorphisms $s_1,\dots , s_{M-1}$ of the vector space *U*(*z*) of *U*-valued rational functions in *M* variables *z* given by

$$ (s_if)(z)=A_{i,U}(s_iz)f(s_iz),\quad i=1,\dots ,M-1, $$

define a representation of the symmetric group $S_M$.

#### Proof

The proof is a straightforward verification that the relations of the symmetric group are equivalent to the identities (i)–(iii) of Theorem 4.1. For example, to check the relation $s_is_{i+1}s_i=s_{i+1}s_is_{i+1}$ we write

$$\begin{aligned} (s_is_{i+1}s_if)(z)&=A_{i,U}(s_iz)(s_{i+1}s_if)(s_iz) \\&=A_{i,U}(s_iz)A_{i+1,U}(s_{i+1}s_iz)(s_if)(s_{i+1}s_iz) \\&=A_{i,U}(s_iz)A_{i+1,U}(s_{i+1}s_iz)A_{i,U}(s_is_{i+1}s_iz)f(s_{i}s_{i+1}s_iz). \end{aligned}$$

We get the left-hand side of (iii) if we replace *z* by $$ s_is_{i+1}s_iz$$ and restrict to the weight space $$U[\mu ]$$. $$\square $$

We notice that the operators $A_{i,U}(z)$ have similar properties as the operators of the dynamical Weyl group of *U* introduced and studied in [7, 24] in the framework of the theory of intertwiners. As in the case of the dynamical Weyl group, their restriction to weight spaces are the image of elements of $U_q\mathfrak {sl}_2$ by embeddings $U_q\mathfrak {sl}_2\hookrightarrow U_q\mathfrak {sl}_M$ corresponding to simple roots,^1^ see [7, Section 4.1]. They obey relations (i) and (iii) of Theorem 4.1 but not (ii). In other words they define a representation of the braid group $B_M$ on rational *U*-valued functions. In this representation the squares $s_i^2$ of the generators act by multiplication by a rational function on each weight space.

Let us compare our operators $A_{i,U}(z)$ with the operators $\mathscr {A}_{s_i,U}(\lambda )$ of [7, Section 4.2] defining the dynamical action of generators, in the case of $U_q\mathfrak {sl}_M$. The latter operators are initially defined as functions of sufficiently large antidominant weights $\lambda$. They extend (upon choosing a logarithm of *q*) to meromorphic functions of $\lambda \in \mathfrak h^*$ in the dual of the Cartan subalgebra $\mathfrak h=\{x\in \mathbb {C}^M\,|\,\sum _i x_i=0\}$ of $\mathfrak {sl}_M$. Let $h_i=(0,\dots ,0,1,-1,0,\dots ,0)$, with 1 in the position $i=1,\dots ,M-1$, be the basis of coroots. To relate *z* to $\lambda$ we identify $\mathfrak h^*$ with $\mathbb {C}^M/\mathbb {C}(1,\dots , 1)$ and write $z=q^{2\lambda }=(q^{2\lambda _1},\dots ,q^{2\lambda _M})$. This is defined up to simultaneous scaling of the variables $z_i$. Since $A_{i,U}(z)$ is a function of the ratios of $z_j$ $A_{i,U}(q^{2\lambda })$ is well-defined.

#### Proposition 4.3

$$ A_{i,U}(q^{2\lambda })|_{U[\mu ]}= {\left\{ \begin{array}{ll} q^{-\mu (h_i)}\mathscr {A}_{s_i,U}(\lambda ), & \text {if } \mu (h_i)\ge 0, \\ \displaystyle (-1)^{\mu (h_i)} q^{-\mu (h_i)}\frac{[\lambda (h_i)-\mu (h_i)/2]_q}{[\lambda (h_i)+\mu (h_i)/2]_q} \mathscr {A}_{s_i,U}(\lambda ),& \text {if } \mu (h_i)\le 0. \end{array}\right. } $$

#### Proof

Since $A_{i,\mu }$ are images of elements of $U_q\mathfrak {sl}_2$ by embeddings into $U_q\mathfrak {sl}_M$, it is sufficient to consider the case $M=2$ and we set $\lambda =\lambda _1-\lambda _2$ and $z=q^{2\lambda }=z_1/z_2$. By complete reducibility we may also assume that $U=L_\ell$ is the irreducible representation of $U_q\mathfrak {sl}_2$ of highest weight $\ell$ and dimension $\ell +1$. It has one-dimensional weight spaces $L_\ell [\ell -2k]$ with basis $v_{\ell -2k}=F^{(\ell )}v_\ell$, ($k=0,\dots ,\ell$), in terms of the highest weight vector $v_\ell$. In this case we have a unique generator $s_1$ sending $x\in \mathfrak h^*$ to $-x$. The following formula for $\mathscr {A}_{s_1,L_\ell }(\lambda )$ can be extracted from [7], see Corollary 8 (iii), Proposition 12 and the definition of the dynamical action of the braid group at the end of Section 4.2:

$$ \mathscr {A}_{s_1,L_{\ell }}(\lambda )v_{m}= (-1)^kq^{m}\prod _{j=1}^{k} \frac{[\lambda +k-j-\ell /2]_q}{[\lambda -k+j+\ell /2]_q}v_{-m},\quad m=\ell -2k. $$

By setting $z=q^{2\lambda }$, this can be rewritten as

$$ \mathscr {A}_{s_1,L_{\ell }}(\lambda )v_{\ell -2k}= (-1)^kq^{m+k(\ell -k+1)}\prod _{j=0}^{k-1}\frac{1-z q^{-\ell +2j}}{1-zq^{\ell -2j}} v_{2k-\ell }. $$

On the other hand, $A_{1,L_\lambda }(z)$ acts on the weight *m* subspace of any representation as $A_m(z)$, see Eq. 10.

It follows from Lemma A.1 that $A_m(z)=\varphi _m(z)\mathscr {A}_{s,L_\ell }(q^{2\lambda })$ with $\varphi _m(z)=q^{-m}$ for $m\ge 0$. If $m\le 0$,

$$ \varphi _m(z) =(-1)^m \prod _{j=\frac{\ell -|m|}{2}}^{\frac{\ell +|m|}{2}-1}\frac{1-zq^{\ell -2j}}{1-zq^{-\ell +2j}} =(-1)^m\frac{1-zq^{-m}}{1-zq^{m}}, \quad m\le 0. $$

so that $$\varphi _m(q^{2\lambda })=(-1)^mq^{-m}\frac{[\lambda -m/2]_q}{[\lambda +m/2]_q}$$. $$\square $$

### Dynamical Weyl Group Action for Symmetrizable Kac–Moody Algebras

Let $U_q\mathfrak g$ be the Drinfeld–Jimbo quantum universal enveloping algebra of a symmetrizable Kac–Moody algebra $\mathfrak g$ with simple roots $\alpha _1,\dots ,\alpha _r$ and simple coroots $h_1,\dots ,h_r$, Cartan matrix $a_{ij}=\alpha _j(h_i)$ and $d_i\in \mathbb {Z}_{\ge 1}$ so that $(d_ia_{ij})$ is symmetric. Let $Q=\oplus _{i=1}^r\mathbb {Z}\alpha _i$ be its root lattice and *T* the torus ${\text {Hom}}(Q,\mathbb {C}^\times )$ of characters of *Q*. The Weyl group $\mathbb {W}$ acts on *R* and *T*. Then for each simple root $\alpha _i$, we have an embedding $j_i:U_{q_i}\mathfrak {sl}_2\rightarrow U_q\mathfrak g$ with $q_i=q^{d_i}$ and an evaluation map $z_{\alpha _i}\in {\text {Hom}}(T,\mathbb {C}^\times )$ so that the ring of regular functions on *T* is $\mathbb {C}[z_{\alpha _1}^{\pm 1},\dots ,z_{\alpha _r}^{\pm 1}]$.

The following generalization of Corollary 4.2, which was formulated as a conjecture in a previous version of this work, is a refinement of the construction of Etingof and Varchenko of a dynamical action of the *braid* group on rational functions with values in an integrable representation. A proof by Anfisa Gurenkova is included in Appendix B.

#### Theorem 4.4

Let *U* be an integrable representation of $U_q\mathfrak g$ and for each $i=1,\dots , r$ let $A_{i,U}(z)$ be the $\textrm{End}(U)$-valued rational function on *T* such that on the weight space of weight $\mu$

$$ A_{i,U}(z)|_{U[\mu ]}=j_i(A_{\mu (h_i)}(z_{\alpha _i})):U[\mu ]\rightarrow U[s_j\mu ], $$

where $A_m(z)$ is defined in Eq. 10. Then the action of simple reflections

$$ (s_if)(z)=A_{i,U}(s_iz)f(s_iz),\quad i=1,\dots r, $$

defines a representation of $\mathbb {W}$ on rational functions on *T* with values in *U*.

### *B*-operators, Quantum Weyl Group, Yangian Limit

The limiting values of *R*-matrices as the spectral parameter tends to 0 or $\infty$ are solutions of the Yang–Baxter equations without spectral parameter. They are *R*-matrices for quantum enveloping algebras of finite dimensional Lie algebras. By dividing the *A*-operators by their limiting values we obtain weight zero “*B*-operators” [7]. Define a formal series $B_m(z)$ by the formula

12 $$\begin{aligned} A_m(z)=A_{m}(0)B_m(z). \end{aligned}$$

The series $B_m(z)$ is well-defined as an endomorphism of the *m*-weight space of any finite dimensional representation and one has a universal formula:

#### Proposition 4.5

(i): For $$m\ge 0$$,
  $$ B_m(z)=\sum _{j=0}^\infty q^{\frac{j(j-3)}{2}}\frac{(-z)^j}{[j]_q!}F^jE^j\prod _{i=1}^j\frac{1-q^2}{1-z q^{m+2i}} $$
(ii): For $$m\le 0$$,
  $$ B_m(z)=\sum _{j=0}^\infty q^{\frac{j(j-3)}{2}}\frac{(-z)^j}{[j]_q!}E^jF^j\prod _{i=1}^j\frac{1-q^2}{1-z q^{-m+2i}} $$

#### Proof

The first formula can be checked directly or deduced from the remark after Corollary 40 in [7], by using Proposition 4.3. There is a similar formula in *loc. cit.*, Proposition 14, but probably the powers of *q* need to be adjusted there. The second formula follows by applying the Cartan involution, see Corollary A.2$\square$

For $m=k-k'$, these operators define via Howe duality $U_q{\mathfrak {gl}}_N$-linear endomorphisms of $\bigwedge ^{k} V(z_1)\otimes \bigwedge ^{k'}V(z_2)$. Their representation theoretical meaning in terms of $U_qL{\mathfrak {gl}}_N$ is not clear but in the limit $q\rightarrow 1$, $A_{m}(0)$ tends up to signs to the permutation *P* and in a suitable $z\rightarrow 1$ limit, $B_m(z)$ converges to a rational *R* matrix, intertwining the coproduct and the opposite coproduct of the Yangian. More precisely we let $q=\exp (\epsilon \hbar )$, $z=\exp (2\epsilon u)$ and consider the limit $\epsilon \rightarrow 0$:

#### Theorem 4.6

Let $\check{R}_{k,k'}(z,q)$ be the family of braiding matrices of Theorem 2.6, viewed as a linear map on the underlying vector space $\bigwedge ^kV\otimes \bigwedge ^{k'}V$. Let

$$ R_{k,k'}(z,q)=P\check{R}_{k,k'}(z,q)\in \textstyle {{\text {End}}_{\mathbb {C}}(\bigwedge ^kV\otimes \bigwedge ^{k'}V)},\text { with } Pv'\otimes v=(-1)^{kk'}v\otimes v', $$

be the corresponding *R*-matrix, normalized to be the identity on the product of highest weight spaces. Then the limit

$$ R^{\textrm{rat}}_{k,k'}(u,\hbar )=\lim _{\epsilon \rightarrow 0}R_{k,k'}(e^{\hbar \epsilon },e^{2\epsilon u}) $$

exists and is a ${\mathfrak {gl}}_N$-invariant solution of the Yang–Baxter equation. Moreover

$$ R^{\textrm{rat}}_{k,k'}(u,\hbar )= \sum _{j\ge 0}\frac{(-\hbar )^j}{j!}\prod _{i=1}^j\frac{1}{u+\hbar (i+|k-k'|/2)} {\left\{ \begin{array}{ll} F^jE^j& \text { if } k\ge k', \\ E^jF^j& \text { if } k\le k'. \end{array}\right. } $$

Here $E=\sum _{i=1}^N\psi ^*_i\otimes \psi _i$, $F=-\sum _{i=1}^N\psi _i\otimes \psi _i^*$ are the $q\rightarrow 1$ limits of the $U_q\mathfrak {sl}_2$-generators of Section 2.

#### Proof

The limit exists since both *E*, *F* and the coefficients in the formula for $\check{R}_{k,k'}$ have a limit as $\epsilon \rightarrow 0$. The Yang–Baxter equation then follows from the braiding relation (4) of $\check{R}_{k,k'}$. The action of the subalgebra $U_q{\mathfrak {gl}}_N$ does not involve the spectral parameter and becomes an action of $U{\mathfrak {gl}}_N$, so that the limit is ${\mathfrak {gl}}_N$-invariant.

By Theorem 4.6$\check{R}_{k,k'}(z,q)$ is a rational function of *z* with poles at finitely many integers powers of *q*. So if we take *q* to be close to 1, $\check{R}_{k,k'}(z,q)$ is holomorphic in, say, the disk $|z|<1/2$. Its value at $q=1$ is independent of *z* and is a homomorphism of $L{\mathfrak {gl}}_N$-modules, which are irreducible for $z=z_1/ z_2\ne 1$. By Schur’s lemma $\check{R}_{k,k'}(z,1)=\check{R}_{k,k'}(0,1)$ must be proportional to the homomorphism $P:u\otimes v\mapsto (-1)^{kk'}v\otimes u$. With the normalization of Theorem 2.6, the factor of proportionality is 1. It follows that

$$ R^{\textrm{rat}}_{k,k'}(u,\hbar ) = \lim _{\epsilon \rightarrow 0}\check{R}_{k,k'}(0,e^{\epsilon \hbar })^{-1} \check{R}_{k,k'}(e^{2\epsilon u},e^{\epsilon \hbar }). $$

By Theorem 2.6 this can be expressed in terms of $A_m(z)=A_m(z,q)$ for $m=k-k'$:

$$ R^{\textrm{rat}}_{k,k'}(u,\hbar ) = \lim _{\epsilon \rightarrow 0}A_m(0,e^{\epsilon \hbar })^{-1}A_m(e^{2\epsilon u},e^{\epsilon \hbar }) = \lim _{\epsilon \rightarrow 0}B_m(e^{2\epsilon u},e^{\epsilon \hbar }). $$

Then, in the limit $\epsilon \rightarrow 0$, $E=\sum \psi _i\otimes \psi _i^*$ and $F=-\sum \psi _i^*\otimes \psi _i$ obey $\mathfrak {sl}_2$ commutation relations and, by Proposition 4.5, $B_m$ tends to

$$ \sum _{j=0}^\infty \frac{(-\hbar )^j}{j!}F^jE^j\prod _{i=1}^j\frac{1}{u+\hbar (i+m/2)} $$

for $$m\ge 0$$ and

$$ \sum _{j=0}^\infty \frac{(-\hbar )^j}{j!}E^jF^j\prod _{i=1}^j\frac{1}{u+\hbar (i-m/2)} $$

for $$m\le 0$$. $$\square $$

#### Remark 4.7

If $k=k'$ we recover the formula proposed by Smirnov, see [21], eq. (115). Understanding this formula was part of the motivation of the present work. For general $k,k'$ our formulas seem to be related to the formula in [21] by a shift of spectral parameter which could be partly explained by a redefinition of exterior powers.

#### Remark 4.8

Another way to arrive at the formula for $R^{\textrm{rat}}$ of Theorem 4.6 is to view it as the *R*-matrix for Yangians and reproduce the proof of Theorem 2.6 in the rational case, by using the Yangian version of the Date–Okado formula (9). A direct proof of the latter is found in [26], see formula (4.14).

## Data Availability

Data sharing not applicable to this article as no datasets were generated or analysed during the current study.

## Appendix A. $U_q\mathfrak {sl}_2$ Yoga

We recall some basic facts about $U_q\mathfrak {sl}_2$, see e.g. [5], and apply them to matrix elements of $A_m(z)$ and $B_m(z)$. The irreducible representations of $U_q\mathfrak {sl}_2$ for *q* not a root of unity are deformations of those of $\mathfrak {sl}_2$. The irreducible representation $L_\ell$ of highest weight $\ell \in \mathbb {Z}_{\ge 0}$ has a basis $v^\ell _\ell ,v^\ell _{\ell -2},\cdots ,v^\ell _{-\ell }$ of weight vectors with $v^\ell _{\ell -2k}=F^{(k)}v^\ell _\ell$.

The Cartan involution is the algebra automorphism of $U_q\mathfrak {sl}_2$ such that $\theta (E)=-F,\theta (F)=-E,\theta (K)=K^{-1}$. It is a coalgebra antihomomorphism and for $v\in L_\ell$, $X\in U_q\mathfrak {sl}_2$, $\theta (X)v=s_\ell X s_\ell ^{-1}v$, where

$$ s_\ell v^\ell _m=(-1)^{(\ell -m)/2}v^\ell _{-m}. $$

Let $C\in Z(U_q\mathfrak {sl}_2)$ be the Casimir element

$$ C=EF+\frac{q^{-1}K+qK^{-1}}{(q-q^{-1})^2}=FE+\frac{qK+q^{-1}K^{-1}}{(q-q^{-1})^2}, $$

belonging to the centre of $U_q\mathfrak {sl}_2$. Its value in $L_\ell$ is

$$ c_\ell =\frac{q^{\ell +1}+q^{-\ell -1}}{(q-q^{-1})^2}. $$

One has the well-known identity, easily checked by induction,

13 $$\begin{aligned} E^jF^j=\prod _{i=0}^{j-1} \left( C-\frac{q^{2i+1}K^{-1}+q^{-2i-1}K}{(q-q^{-1})^2} \right) . \end{aligned}$$

### Lemma A.1

Let $A_m(z)$ be the formal power series (10) and $B_m(z)$ the formal power series defined by Eq. 12. Then

$$\begin{aligned} A_m(z)v_m^\ell&=\prod _{j=0}^{\frac{\ell -|m|}{2}-1} \frac{z- q^{\ell -2j}}{1-z q^{\ell -2j}}v_{-m}^\ell \\ B_m(z)v_m^\ell&=\prod _{j=0}^{\frac{\ell -|m|}{2}-1} \frac{1-z q^{-\ell +2j}}{1-z q^{\ell -2j}}v_{m}^\ell \end{aligned}$$

### Proof

Let us first consider the case $m\ge 0$.

The identity Eq. 13 implies that on the subspace of $L_\ell$ of weight $-\ell +2k$, we have

$$\begin{aligned} E^jF^j|_{L_{\ell }[-\ell +2k]}&=\prod _{i=0}^{j-1} \frac{q^{\ell +1}+q^{-\ell -1}-q^{2i+1+\ell -2k}-q^{-2i-1-\ell +2k}}{(q-q^{-1})^2} \\&=\prod _{i=0}^{j-1} \frac{(q^{\ell +1-k+i}-q^{-\ell -1+k-i})(q^{k-i}-q^{-k+i})}{(q-q^{-1})^2} \\&=\prod _{i=0}^{j-1} [\ell +1-k+i]_q[k-i]_q. \end{aligned}$$

It follows that for $m=\ell -2k\ge 0$

$$ A_m(z)=\sum _{j=0}^{k}(-q)^j\frac{1-q^mz}{1-q^{2j+m}z}\frac{1}{[j]_q![j+m]_q!} \prod _{i=0}^{j-1}[\ell +1-k-i]_q[k-i]_qF^m $$

Therefore,

$$\begin{aligned} A_m(z)v^\ell _{m}&= \sum _{j=0}^{k}(-q)^j\frac{1-q^mz}{1-q^{2j+m}z}\frac{[\ell -k]_q!}{[j]_q![j+m]_q![k]_q!} \prod _{i=0}^{j-1}[\ell +1-k-i]_q[k-i]_qv^\ell _{-m} \\&= \sum _{j=0}^{k}(-q)^j\frac{1-q^{\ell -2k}z}{1-q^{2j+\ell -2k}z}\frac{\prod _{i=0}^{k-1}[\ell -k+j-i]_q}{[j]_q![k-j]_q!}v^\ell _{-m}. \end{aligned}$$

We thus have a rational function of *z* with simple poles at $z=q^{-\ell +2k-2},$ $q^{-\ell +2k-4},$ $\dots ,q^{-\ell }$ which is bounded at infinity. By comparing the residues at the poles and the value $\genfrac[]{0.0pt}1{\ell -k}{k}_q$ at $z=q^{-\ell +2k}$, we see that

$$ A_m(z)v^\ell _m= \prod _{j=0}^{k-1}\frac{z-q^{\ell -2j}}{1-zq^{\ell -2j}}v^\ell _{-m},\quad \text {if } m=\ell -2k\ge 0. $$

For $m\le 0$, the inversion relation implies that $A_{m}(z)v^\ell _m=A_{|m|}(z^{-1})^{-1}v^\ell _{m}$. With $-m=\ell -2k\ge 0$ we find that

$$\begin{aligned} A_m(z)v^\ell _m&=\prod _{j=0}^{k-1} \frac{1-z^{-1}q^{\ell -2j}}{z^{-1}-q^{\ell -2j}}v^\ell _{-m} \\&= \prod _{j=0}^{k-1}\frac{z-q^{\ell -2j}}{1-zq^{\ell -2j}}v^\ell _{-m}, \quad \text {if } -m=\ell -2k\ge 0. \end{aligned}$$

This proves the formula for $A_m(z)$ since $k=\frac{\ell -|m|}{2}$. In particular, $A_m(0)v^\ell _m=\prod _{j=0}^{k-1}(-q^{\ell -2j})v^\ell _{-m}$, and the formula for $B_m(z)=A_m(0)^{-1}A_m(z)$ follows. $\square$

### Corollary A.2

Let *U* be any finite dimensional representation of $U_q\mathfrak {sl}_2$. Then for any $m\in \mathbb {Z}$

$$ \theta (B_m(z))|_{U[-m]}=B_{-m}(z)|_{U[-m]} $$

### Proof

It is sufficient to prove this for *U* irreducible. In this case the statement follows from Lemma A.1 and the identity $\theta (X)=s_\ell X s_\ell ^{-1}$ for the action of $X\in U_q\mathfrak {sl}_2$ on $L_\ell$. $\square$

## Appendix B. Proof of Theorem 4.4, by Anfisa Gurenkova

We use the notations of Section 4.2 and adopt the conventions of [7]. We denote by $\mathbb {W}$ and $\tilde{\mathbb {W}}$ the Weyl group and the braid group of the root system of the symmetrizable Kac–Moody algebra $\mathfrak g$, respectively. For each element $w\in \mathbb {W}$ and an integrable $U_q\mathfrak {g}$-module *V* Etingof and Varchenko [7] define an operator $\mathscr {A}_{w, V}(\lambda , \mu ): V[\mu ]\rightarrow V[w\mu ]$. The following is true:

### Proposition B.1

(Lemma 17 in [7]) If $l(w_1 w_2)=l(w_1)+l(w_2)$, then

14 $$\begin{aligned} \mathscr {A}_{w_1w_2, V}(\lambda , \mu ) = \mathscr {A}_{w_1, V}(w_2\lambda , w_2\mu ) \mathscr {A}_{w_2, V}(\lambda , \mu ) \end{aligned}$$

The definition of the operators $\mathscr {A}_w$ is consistent with restricting to the root $\mathfrak {sl}_2$ in the following sense (see [7, Section 4.1]).

### Proposition B.2

For any *i*:

15 $$\begin{aligned} \mathscr {A}_{s_i, V}(\lambda , \mu ) = \mathscr {A}_{s, V'}(\lambda (h_i),\mu (h_i)), \end{aligned}$$

where $V'$ is the $U_{q_i}(\mathfrak {sl}_2)$-module pulled back by the embedding $j_i$.

Due to these two facts, it is enough to construct the operators $\mathcal {A}_{s,V}(l,m)$ for $U_q\mathfrak {sl}_2$-modules *V*. Then one defines an operator $\mathscr {A}_{w,V}$ by decomposing *w* into simple reflections (14) and then using Eq. 15. Also one can define $\mathscr {A}_{w,V}$ for any $w\in \tilde{\mathbb {W}}$ by this procedure.

By Proposition B.1, the operators $\mathscr {A}_{w, V}$ define an action of $\tilde{\mathbb {W}}$ on *V*-valued functions of $\lambda$ as follows:

16 $$\begin{aligned} (w * u)(\lambda ) = \mathscr {A}_{w,V}(w^{-1}(\lambda )) u(w^{-1}\lambda ). \end{aligned}$$

Let $P\tilde{\mathbb {W}}$ be the pure braid group, i.e. the kernel of the map $\tilde{\mathbb {W}}\rightarrow \mathbb {W}$. It is a normal subgroup in $\tilde{\mathbb {W}}$ generated by $s_i^2$. Notice that $\mathscr {A}_{s_i}(s_i\lambda ,s_i\mu )\mathscr {A}_{s_i}(\lambda ,\mu )$ is a weight zero operator. Moreover, it is diagonal in a basis of $V[\mu ]$ which is consistent with the decomposition of *V* into irreducible $j_i(U_{q_i}(sl_2))$-modules. We will see in Lemma B.5 that in fact it acts by a scalar (depending on $\lambda$, $\mu$, and *q*). Therefore it defines a character $\chi _V: P\tilde{\mathbb {W}} \rightarrow Fun(\lambda ,\mu ,q)$ with values in the meromorphic functions in $\lambda$, $\mu$, and *q*.

Our goal is to modify the action Eq. 16 by multiplying each $\mathscr {A}_w(\lambda ,\mu )$ by a suitable function of $\lambda , \mu , q$ so that it factors through $\mathbb {W}$.

First, we find the type of functions which do not break the braid relation. Second, we compute the character and see that it belongs to this type of functions.

### Lemma B.3

Let

$$ \tilde{\mathscr {A}_{s_i}}(\lambda ,\mu ) = f(\lambda (h_i), \mu (h_i),q_i )\mathscr {A}_{s_i}(\lambda ,\mu ) $$

for some function *f*(*l*, *m*, *q*). Then the operators $\tilde{\mathscr {A}_{s_i}}(\lambda ,\mu )$ satisfy the braid relations.

### Proof

For any two nodes 1,2 of the Dynkin diagram consider the corresponding simple reflections $s_1, s_2$. Either there is no relation between them, or they satisfy the braid relation $s_i\cdots s_2 s_1 s_2 = s_j\cdots s_1 s_2 s_1$. Here the number of factors on both sides is equal to some integer $m_{12}$, depending on the number of edges between the nodes 1 and 2, and $(i,j)=(1,2)$ or (2, 1) depending on the parity of $m_{12}$. Denote this product by *w*. Since $l(w) = m_{12}$, by Proposition B.1 we have

17 $$\begin{aligned}  &   \mathscr {A}_{s_i, V}(s_j\cdots s_2(\lambda , \mu ))\cdots \mathscr {A}_{s_1, V}(s_2(\lambda , \mu )) \mathscr {A}_{s_2, V}(\lambda , \mu ) = \nonumber \\  &   \qquad \qquad \qquad \qquad \quad = \mathscr {A}_{s_j, V}(s_i\cdots s_1(\lambda , \mu ))\cdots \mathscr {A}_{s_2, V}(s_1(\lambda , \mu )) \mathscr {A}_{s_1, V}(\lambda , \mu ) \end{aligned}$$

When we replace $\mathscr {A}$-s by $\tilde{\mathscr {A}}$-s in the LHS of Eq. 17, it is multiplied by

$$ f\Big ( s_j\cdots s_2(\lambda , \mu )(h_i),q_i\Big )\cdots f\Big ( s_2(\lambda , \mu )(h_1),q_1\Big ) f\Big ((\lambda , \mu )(h_2),q_2\Big ), $$

The following fact applied to a subalgebra corresponding to the pair of roots $\alpha _1,\alpha _2$ finishes the proof:

### Lemma B.4

Let $\mathfrak {g}$ be a finite-dimensional Lie algebra of rank 2 and $w_0=s_{i_1}\cdots s_{i_l}$ be a reduced word for the longest element in $\mathbb {W}$. Then for any weight $\lambda$ the set {$\big (s_{i_{k+1}}\cdots s_{i_l}\lambda (h_{i_k}),d_{i_k}\big )| k=1\ldots l-1$} does not depend on a reduced word.

### Proof

It is a reformulation of Lemma 2 in [7]. For reader’s convenience we reproduce its proof here.

Let $(\, , )$ be the non-degenerate bilinear form on $\mathfrak {h}$ such that $(h, h_i) = d^{-1}_i\alpha _i(h)$. Notice that $s_{i_{k+1}}\cdots s_{i_l}\lambda (h_{i_k}) = \lambda (s_{i_l}\cdots s_{i_{k+1}} h_{i_k})$. Denote $h^k:= s_{i_l}\cdots s_{i_{k+1}} h_{i_k}$. Also notice that $d_i = \frac{2}{(h_i, h_i)} = \frac{2}{(wh_i,wh_i)}$ for any $w\in \mathbb {W}$. Since $w_0$ is the longest element, $\{h^k\}$ is the set of all the positive coroots, each appearing exactly once. Therefore the set in the statement is $\{\big (\lambda (h^l), \frac{2}{(h^l,h^l)}\}$ which does not depend on a reduced decomposition. $\square$

### Lemma B.5

The character $\chi _{V}: P\tilde{\mathbb {W}} \rightarrow Fun(\lambda ,\mu ,q)$ is defined by

$$ \chi _{V}(s_i^2) = (-1)^{\mu (h_i)}\frac{[(\lambda +\frac{\mu }{2})(h_i)]_{q_i}}{[(\lambda -\frac{\mu }{2})(h_i)]_{q_i}} $$

### Proof

By the consistency with the restriction (15) it is enough to prove the statement for $U_q\mathfrak {sl}_2$. Let $V_n$ denote the irreducible $\mathfrak {sl}_2$-module of the highest weight *n*. By corollary 8, Proposition 12 and the definitions of section 4.2 of [7],

$$ \mathscr {A}_{s, V_n[n-2k]}(l, n-2k) = A^\infty _{V_n[n-2k]} B_{V_n[n-2k]}, $$

where $A^\infty _{V_n[n-2k]}v_{n-2k} = (-1)^kq^{n-2k}v_{2k-n}$ and $B_{V_n}$ is a weight zero operator defined by

$$ B_{V_n[n-2k]}(l) = \prod _{j=1}^k\frac{[l+1+j]_q}{[l+1-n+2k-j]_q}. $$

Therefore for $$m=n-2k$$

$$\begin{aligned} \chi _{V_n}(s^2)(l,m)&= \mathscr {A}_{s, V_n}(-l,-m) \mathscr {A}_{s, V_n}(l, m) \\&= B_{V_n[-m]}(l-1+\frac{-m}{2}) B_{V_n[m]}(-l-1+\frac{m}{2})\cdot (-1)^n \\&=(-1)^m\prod _1^{n-k}\frac{[l+\frac{-n+2k}{2}+j]_q}{[l-\frac{-n+2k}{2}-j]_q}\prod _1^k\frac{[-l+\frac{n-2k}{2}+j]_q}{[-l-\frac{n-2k}{2}-j]_q}\\&= (-1)^m\frac{\prod _{k+1}^{n}[l-\frac{n}{2}+j]_q\prod _0^{k-1}[l-\frac{n}{2}+j]_q}{\prod _{k+1}^{n}[l+\frac{n}{2}-j]_q\prod _0^{k-1}[l+\frac{n}{2}-j]_q} \\&=(-1)^m\prod _{-\frac{n}{2}}^{\frac{n}{2}}\frac{[l+j]_q}{[l-j]_q}\cdot \frac{[l+\frac{n}{2}+k]_q}{[l-\frac{n}{2}-k]_q} = (-1)^n\frac{[l+\frac{m}{2}]_q}{[l-\frac{m}{2}]_q} \end{aligned}$$

$$\square $$

### Proposition B.6

Let

$$ A_{s_i, V[\mu ]} = {\left\{ \begin{array}{ll} q_i^{-\mu (h_i)}\mathscr {A}_{s_i,V[\mu ]}, &  \text {if } \mu (h_i)\ge 0, \\ (-1)^{\mu (h_i)}q_i^{-\mu (h_i)}\frac{[(\lambda -\frac{\mu }{2})(h_i)]_{q_i}}{[(\lambda +\frac{\mu }{2})(h_i)]_{q_i}}\mathscr {A}_{s_i, V[\mu ]}, &  \text {if } \mu (h_i)< 0\end{array}\right. } $$

Then $A_{s_i}$ define the action of the Weyl group on $V-$valued functions of $\lambda$ via formulas (14) and (16).

### Proof

From Lemma B.3 it follows that $A_{s_i}$ satisfy the braid relations. It suffices to check the relation $(s_i*)^2 = 1$ for $U_q\mathfrak {sl}_2$. In this case

$$ A_{s, V_n}(-l,-m)A_{s,V_n}(l,m) = (-1)^n\frac{[-l+\frac{m}{2}]_q}{[-l-\frac{m}{2}]_q} \cdot \chi _{V_n}(s^2)(l,m) = 1 $$

$$\square $$

### Remark B.7

In the case $\mathfrak {g}=\mathfrak {sl}_n$ the operators $A_{s_i, V[\mu ]}$ coincide with the operators defined in Proposition 4.3.

## Footnotes

1 In [7] the dynamical Weyl group is defined for any Kac–Moody Lie algebra.
